# Childhood trauma is associated with age at onset of symptoms, functioning, and cognition in patients with schizophrenia

**DOI:** 10.47626/2237-6089-2019-0081

**Published:** 2022-06-14

**Authors:** Emmanuely Macedo Santana De-Nardin, Camilla Alves Muratori, Isabela Serra Ribeiro, Rodrigo Barreto Huguet, Joao Vinicius Salgado

**Affiliations:** 1 Programa de Pós-Graduação em Neurociências Instituto de Ciências Biológicas Universidade Federal de Minas Gerais Belo Horizonte MG Brazil Programa de Pós-Graduação em Neurociências, Instituto de Ciências Biológicas, Universidade Federal de Minas Gerais (UFMG), Belo Horizonte, MG, Brazil.; 2 Fundação de Amparo à Pesquisa do estado de Minas Gerais Belo Horizonte MG Brazil Fundação de Amparo à Pesquisa do estado de Minas Gerais (FAPEMIG), Belo Horizonte, MG, Brazil.; 3 Instituto de Previdência dos Servidores do Estado de Minas Gerais Belo Horizonte MG Brazil Instituto de Previdência dos Servidores do Estado de Minas Gerais, Belo Horizonte, MG, Brazil.; 4 Departamento de Morfologia Instituto de Ciências Biológicas UFMG Belo Horizonte MG Brazil Departamento de Morfologia, Instituto de Ciências Biológicas, UFMG, Belo Horizonte, MG, Brazil.

**Keywords:** Childhood trauma, cognition, functioning, schizophrenia

## Abstract

**Introduction:**

Childhood trauma (CT) is known to be a vulnerability factor for schizophrenia, but the specific impacts of different trauma subtypes on the prognosis of these patients remains unclear.

**Objective:**

To assess the relationships between the occurrence of overall CT and its subtypes with factors with known prognostic impact on schizophrenia, such as age at onset of symptoms, global functioning, and cognitive impairment in a sample of Brazilian patients.

**Methods:**

One hundred and five stable patients diagnosed with schizophrenia according to DSM-5 criteria were evaluated using the Independent Living Skills Survey (ILSS; self-report global functioning), Schizophrenia Cognition Rating Scale (SCoRS; subjective cognitive impairment), and Childhood Trauma Questionnaire scales (CTQ; perceived overall CT, emotional neglect, physical neglect, physical abuse, and emotional and sexual abuse). Statistical analysis was performed with multivariate linear regression.

**Results:**

After controlling for educational level and age, subjective cognitive impairment was directly correlated with overall perceived CT occurrence, emotional abuse, and sexual abuse. Self-report global functioning was inversely correlated with perceived overall CT occurrence, emotional abuse, and sexual abuse. Emotional abuse and physical abuse were also inversely correlated with age at onset of symptoms.

**Conclusions:**

CT can be related to more severe prognoses in schizophrenia, impacting on early onset of symptoms, lower global functioning, and greater cognitive impairment. Subtypes of trauma can be associated with different prognostic risks.

## Introduction

The neurodevelopmental hypothesis of schizophrenia refers to a complex interaction between risk and vulnerability factors. A better understanding of these factors is fundamental to enable early interventions in patients with this disease.^1^

Childhood trauma (CT) is a well-documented risk factor for schizophrenia, in association with genetic and other environmental factors.^2^ CT has been associated with severity of symptoms ^3-5^ and with cognitive and functional performance^6-13^ in patients with schizophrenia.

In a recent review, Popovic et al.^14^ showed associations between CT and poor performance in patients with schizophrenia and subjects at high-risk of psychosis. The cognitive functions most affected by CT were working memory, executive functions, and attention.^14,15^ CT has also been associated with young age at onset of psychotic symptoms.^16^

Other aspects associated with CT in schizophrenia include inflammatory biomarkers,^17,18^ body-mass index,^19^ telomere length,^19^ hair cortisol concentration,^20^ suicide,^21,22^ violence,^23^ insight,^24^ quality of life,^25^ social cognition,^26^ and social functioning.^27,28^

Most of the earlier research on CT and schizophrenia accounted for a heterogeneous concept of general trauma without considering its different subtypes, such as physical abuse, emotional abuse, sexual abuse, physical neglect and emotional neglect. It has been shown that these subtypes may impact differently on prognostic parameters of patients with schizophrenia^29^ but, to date, evidence about these differences is still scarce and somewhat divergent. While some studies have indicated that emotional abuse has a greater impact, others have not found any difference between subtypes.^30-34^ It is noteworthy that recent evidence indicates that sexual abuse plays a less prominent role than initially supposed.^5,35^ Furthermore, it is possible that the relevance of CT subtypes varies between different countries and cultures, due to differences in socioeconomic aspects including the ways children are treated or educated. To our knowledge, there is no data regarding the Brazilian population, which may be considerably different from populations analyzed in existing similar studies (e.g., American, European, and East Asian).

This study aims to contribute to better elucidating the associations between CT (in general and its subtypes) and prognostic parameters, such as age at onset of symptoms, cognition, and functionality, in a sample of Brazilian patients with schizophrenia.

## Methods

### Participants

The sample was randomly recruited from patients from three Primary Care Network and Specialty Clinics services in the towns of Belo Horizonte, Contagem, and Nova Lima, all in the state of Minas Gerais, Brazil. Enrollment took place between September 2015 and January 2017.

Patients aged between 18 and 60 years and diagnosed with schizophrenia according to the Diagnostic and Statistical Manual of Mental Disorders (DSM-5) criteria and confirmed by the Mini International Neuropsychiatric Interview (MINI-Plus) were included. We excluded patients that met the following criteria: 1) illiteracy or clear difficulty with filling out the research instruments; 2) any current psychiatric comorbidity identified by the MINI-Plus, including Mood Disorders and Substance Use Disorders; 3) diagnoses of neurological diseases; or 4) patients who were clinically unstable at the time of the interview, defined as a score above 19 on the positive symptoms subscale (or a score above 4 on any item) of the Positive and Negative Syndrome Scale (PANSS).

The study was approved by the ethics committee at Fundação Hospitalar do Estado de Minas Gerais (FHEMIG) and all participants signed a written consent form after receiving detailed information about the procedure.

### Measures

Sociodemographic and clinical data were collected using a standard questionnaire designed for the study. Age at onset of symptoms was categorized as early childhood and adolescence or early adulthood (≥ 18 years) and analyzed as a covariate.

#### Perceived CT

To evaluate the occurrence of traumas in childhood, we used the short version of the Childhood Trauma Questionnaire (CTQ), translated and validated for the Brazilian context.^36,37^ This questionnaire can identify five components of trauma: physical abuse, emotional abuse, sexual abuse, physical neglect, and emotional neglect. We also obtained a total score corresponding to overall occurrence of CT. The scale is self-administered and the interviewer’s intervention was limited to providing clarification, only when requested by the patient.

#### Self-report functioning

A validated Brazilian version of the Independent Living Skills Survey (ILSS)^38^ was used to assess independent living skills in several areas of functioning in everyday life, including appearance and clothing, personal hygiene, personal care, food, health, money, transportation, entertainment, and employment. The patient’s level of functioning is evaluated in each area and with a total score.^38,39^ In our study, we chose to use this last criterion, so as to analyze the patient’s global functioning.

#### Subjective cognition

We used a validated Brazilian version of the Schizophrenia Cognition Rating Scale (SCoRS) to assess cognition.^40,41^ This instrument was designed for patients with schizophrenia and is able to evaluate cognitive dimensions such as attention, memory, reasoning, ability to solve problems, working memory, language, and motor skills. Cognition is assessed with 20 questions, with scores ranging from 1 to 4. The higher the score obtained, the greater the cognitive deficit observed. The degree of cognitive impairment corresponds to the sum of all 20 scores (total score). The SCoRS has very good psychometric properties, including convergent validity as measured by comparison with the Brief Assessment of Cognition in Schizophrenia (BACS).^41^

## Design and statistical analysis

All patients completed the sociodemographic and clinical questionnaires, CTQ, SCoRS, and ILSS. To describe the sample, we used frequency distribution tables to analyze categorical variables and measures of central tendency, position, and variability to analyze numerical variables.

We tested associations between CTQ (overall and subtypes) and outcome variables: onset of symptoms, SCoRS total score, and ILSS total score, respectively. We chose to use non-parametric tests, because most of the variables had asymmetric distribution, as analyzed by the Kolmogorov-Smirnov test. Spearman’s correlation coefficients were used to assess associations with numerical variables (age at onset of symptoms and the ILSS and SCoRS scores along with the results of the CTQ scale). The Mann-Whitney test was used for comparisons between groups.

After the univariate analyses, multivariate analyses were conducted to determine the influence of age and educational level on associations between perceived CT with variables of interest. We estimated two separate models for each outcome variable: 1) A model with total CTQ score, age, and educational level, and 2) a model with all CT subtypes, age, and educational level.

We used the linear regression model for the multivariate analyses, adopting a *p* value less than 0.20 in the univariate analysis as inclusion criterion. We removed variables from the model using the backward criterion, maintaining a significance level of 5%. The adjustment of the final model was evaluated by estimating the coefficient of determination (R^2^) and examining diagnostic plots. Considering the possibility of collinearity between the results for CT subtypes and CTQ total scores, a multivariate analysis of the final model was also estimated with separate scales, but did not indicate significant changes in adjustment estimates (R^2^).

All analyses were performed using the SPSS statistical package, version 19.0, adopting a 5% significance level for all operations.

## Results

### Sociodemographic and clinical data

A total of 105 patients with schizophrenia participated in the study, 57.1% of whom were male and 42.9% of whom were female, with a mean age of 40.2 years. The mean age at onset of symptoms was 24.3 (±9.7 years). For 68.6% of the patients, symptoms started during childhood or adolescence (i.e. at an age younger than 18 years). Table 1 shows the data on age at symptom onset and the scores on the scales used.

**Table 1:** Descriptive analysis of age at onset of symptoms and scores on the ILSS-BR/P, SCoRS-BR, and CTQ scales

	Mean	SD	Median	Minimum	Maximum
Age at onset of symptoms	24.3	9.7	21.0	5.0	63.0
Total ILSS-BR/P	71.4	13.9	72.0	39.0	94.0
Total interviewer – SCoRs-BR	39.2	13.1	36.0	19.0	74.0
CTQ physical neglect	6.4	2.2	6.0	3.0	13.0
CTQ emotional neglect	16.4	6.1	15.0	7.0	31.0
CTQ emotional abuse	10.9	5.0	10.0	5.0	25.0
CTQ physical abuse	8.5	3.4	8.0	5.0	23.0
CTQ sexual abuse	9.9	6.2	7.0	5.0	25.0
Total CTQ	52.1	16.2	47.0	27.0	95.0

CTQ = Childhood Trauma Questionnaire; ILSS-BR/P = Independent Living Skills Survey, Brazilian Portuguese Patient version; SCoRs-BR = Schizophrenia Cognition Rating Scale, Brazilian Portuguese version; SD = standard deviation.

### Association between perceived CT and age at onset of symptoms

The age at onset of symptoms was significantly associated with the emotional neglect, emotional abuse, physical abuse, and sexual abuse subscales and with the total score of the CTQ scale (p < 0.05). Patients whose symptoms started before the age of 18 years had higher mean scores on these subscales and on the total CTQ scale when compared to patients whose symptoms began in adulthood. Correlations are shown in Table 2.

**Table 2:** Correlations between CTQ scale results, age at onset of symptoms, and scores on the ILSS-BR/P and SCoRS-BR scales

CTQ	Age at onset of symptoms		ILSS-BR/P		SCoRS-BR – Total interviewer	
	Spearman coefficient	p-value	Spearman coefficient	p-value	Spearman coefficient	p-value
Physical neglect	-0.145	0.139	-0.250	0.010	0.212	0.030
Emotional neglect	-0.218	0.025	-0.338	< 0.001	0.182	0.063
Emotional abuse	-0.388	< 0.001	-0.362	< 0.001	0.393	< 0.001
Physical abuse	-0.232	0.017	-0.179	0.067	0.313	0.001
Sexual abuse	-0.260	0.007	-0.321	0.001	0.380	< 0.001
Total CTQ	-0.356	< 0.001	-0.444	< 0.001	0.422	< 0.001

CTQ = Childhood Trauma Questionnaire; ILSS-BR/P = Independent Living Skills Survey, Brazilian Portuguese Patient version; SCoRS-BR = Schizophrenia Cognition Rating Scale, Brazilian Portuguese version.

In the multivariate analysis, however, only CTQ total score and the emotional abuse and physical abuse subtypes remained significantly associated with age at symptoms onset, after controlling for age and educational level, as shown in Table 3.

**Table 3:** Final model evaluating factors associated with the CTQ scale (subtypes and total)

	Coefficient [95%CI] and p-value*		
	SCoRs scale	ILSS scale	Age at onset of symptoms
Model with subtypes			
Emotional abuse	1.12 [0.71 to 1.52]; p < 0.001	-1.04 [-1.50 to -0.58]; p < 0.001	-0.41 [-0.73 to -0.09]; p = 0.012
Physical abuse			-0.57 [-1.05 to -0.09]; p = 0.020
Sexual abuse	0.50 [0.16 to 0.84]; p = 0.004	-0.37 [-0.76 to 0.00]; p=0.054	
	**F = 32.08 (p < 0.001)**	**F = 23.97 (p < 0.001)**	**F = 19.281 (p < 0.001)**
Adjusted R^2^	47.3%	39.9%	35.1%
Model with total CTQ	0.43 [0.30 to 0.55]; p < 0.001	-0.38 [-0.52 to -0.25]; p < 0.001	-0.18 [-0.27 to -0.08]; p < 0.001
	**F = 25.97 (p < 0.001)**	**F=23.69 (p < 0.001)**	**F = 8.677 (p < 0.001)**
Adjusted R^2^	41.9%	39.6%	34.5%

Multivariate analysis used the linear regression model, in which variables with a p-value less than 0.20 were included in the univariate analysis. The backward criterion was used for the exclusion of variables from the final model. These adjustments were evaluated by means of estimating the coefficient of determination (R^2^) and diagnostic plots. We adopted a significance level of 5% for all variables.

95%CI = 95% confidence interval; CTQ = Childhood Trauma Questionnaire; ILSS = Independent Living Skills Survey; SCoRs = Schizophrenia Cognition Rating Scale.

* Values adjusted by age and educational level.

### Association between perceived CT and self-reported functioning

The physical neglect, emotional neglect, emotional abuse, and sexual abuse subscales and the total CTQ score all had significant inverse correlations with the score on the ILSS scale. All of these correlations are shown in Table 2.

In the multivariate analysis, however, only CTQ total score and the emotional abuse subtype remained significantly associated with self-reported functioning, after controlling for age and educational level. The Sexual Abuse subtype remained marginally significant (p = 0.054). These results are shown in Table 3.

### Association between CT and subjective cognitive impairment

Physical neglect, physical abuse, emotional abuse and sexual abuse - and the total score on the CTQ scale all had significant direct correlations with the SCoRS results. All these correlations are shown in Table 2.

In the multivariate analysis, however, only CTQ total score and the emotional abuse and sexual abuse subtypes remained significantly associated with age at symptoms onset, after controlling for age and educational level, as shown in Table 3.

## Discussion

The median CTQ scores of our sample indicated low to moderate perceived CT, overall and for all of its subtypes, according to the cutoff points used for this scale.^16^ Subjective cognitive impairment was very similar to results reported in previous studies using the SCoRS,^41^ including studies in Brazil.^40^ The mean age at onset of symptoms of our sample and the performance on ILSS were also comparable to those from similar studies.^16,42^

We found significant associations between perceived CT occurrence and prognostic outcomes in adult patients with schizophrenia. After controlling for educational level and age, the emotional abuse subtype was the most frequent, whereas emotional and physical neglect were the least significant subtypes. This highlights the importance of abuse traumas in the clinical picture of psychotic disorders in Brazil.

The association of the emotional abuse subtype with young age at onset of symptoms corroborates a recent study conducted in China.^43^ The only other study that investigated the association between age at onset of symptoms and CTQ was conducted in Turkey and reported that all types of trauma except for physical abuse were found to be related to earlier onset of symptoms. However, that study did not conduct a multivariate analysis.^16^ Altogether, these results corroborate the hypothesis that emotional abuse is associated with worse prognosis and clinical severity in schizophrenia.^5,35^

To our knowledge, we are the first to investigate associations between CT and self-reported real-world functioning as measured by ILSS in patients with schizophrenia. Some recent studies have used a similar approach investigating the association between perceived CT and social functioning. In Norway, Hjelseng et al.^28^ found that the severity of CT was inversely associated with scores on a social functioning scale, but their study did not control for educational level or age. The CT subtypes associated with social functioning were emotional and physical abuse, and emotional neglect. In Korea, Kim et al.^27^ showed that after controlling for sex and age, emotional neglect significantly predicted the score on a social functioning scale in patients with schizophrenia. The discrepancy between our findings and the literature suggests that CT subtypes may differ between countries or cultures as predictors of real world functioning.

Subjective cognition assessed by the SCoRS was associated with overall perceived CT and the physical neglect, physical abuse, emotional abuse, and sexual abuse subtypes. All other studies investigating perceived CT subtypes and cognitive impairment in schizophrenia used neuropsychological tests rather than cognitive scales. These studies found more specific associations between subtypes of CT and subtypes of cognitive impairment. One study categorized CT into two subtypes, abuse and neglect, and found that neglect was a significant predictor of impairment in social cognition and verbal learning among Norwegian patients.^44^ Another study found that physical neglect was associated with working memory and attention in South African patients.^15^ Besides cultural differences, one possible explanation for the discrepancies between these results and ours is that subjective cognitive scales like SCoRS could be more sensitive but less specific than neuropsychological tests for detecting the impact of trauma on cognition.

In sum, our sample of patients with schizophrenia showed significant correlations between the occurrence of perceived CTs and lower self-reported global functioning, greater subjective cognitive impairment, and younger age at onset of symptoms. Some subtypes of abuse trauma were specifically associated with some prognostic aspects, such as emotional abuse with all outcome measures, physical abuse with age at onset of symptoms, and sexual abuse with self-reported functioning. The discrepancy between our findings and those from other countries regarding the relative weight of the nature of the trauma as a predictor of schizophrenia outcomes is noteworthy. In several studies from developed countries, trauma of the neglect subtype is preponderant, although abuse (mainly emotional) is also important in some studies. Further studies are required to confirm the hypothesis that sociocultural factors influence the associations between trauma subtype and outcome in schizophrenia.

Our study has strengths, such as multicenter recruitment of patients, yielding a varied sample, and exclusion of non-stabilized patients that could otherwise introduce bias, impairing the results. We only used validated instruments and we sought to explore the subtypes of traumas in childhood, rather than being restricted to reporting the occurrence of traumas in general. At present, the CTQ is the instrument most used worldwide to assess CT and its subtypes, allowing for direct comparisons with the literature.^14^ To our knowledge, this was the first time that associations have been investigated between CT and scales of real-world functioning (such as the ILSS) and cognitive performance (the SCoRS). Functioning and cognition are very important dimensions in patients’ prognoses, but data are still scarce within this approach.

Our study also has limitations: the CTQ does not allow the subjective nature of the trauma to be assessed, as there is possible memory bias in a retrospective approach and it is possible that cognitive impairment and medication use could influence the patients’ responses. However, as pointed out by Popovic et al.^14^ this kind of limitation may be unavoidable, due to the very nature of CT. In retrospective studies, the only means of assessing CT is by using self-report measures and the influence of memory bias, cognitive impairment, and medication use is also well-documented. The same limitation inherent to patient self-reports also apply to the other scales used - SCoRS and ILSS.

In conclusion, CT is related to worse prognostic impact in patients with schizophrenia and is mainly related to onset of symptoms during childhood and adolescence, impaired global functioning, and greater cognitive impairment. Different subtypes of trauma seem to influence these outcomes differently, although these associations need to be more deeply investigated in prospective studies.
